# Fishing for drugs

**DOI:** 10.7554/eLife.76632

**Published:** 2022-02-08

**Authors:** Chinyere Kemet, Emily Hill, Hui Feng

**Affiliations:** 1 Department of Pharmacology and Experimental Therapeutics, Boston University School of Medicine Boston United States; 2 Department of Medicine, Section of Hematology and Medical Oncology, Boston University School of Medicine Boston United States

**Keywords:** metastasis, gastrulation, cancer, phenotyping screening, epiboly, Pizotifen, Zebrafish, Mice

## Abstract

Screening for drugs that disrupt embryonic development in zebrafish can help identify treatments that suppress metastasis.

**Related research article** Nakayama J, Tan L, Li Y, Goh BC, Wang S, Makinoshima H, Gong Z. 2021. A zebrafish embryo screen utilizing gastrulation identifies the HTR2C inhibitor Pizotifen as a suppressor of EMT-mediated metastasis. *eLife* **10**:e70151. doi: 10.7554/eLife.70151

About 90% of all cancer-related deaths are caused by metastasis, which is when cancer cells spread to other parts of the body to form new tumors (Chaffer and Weinberg, 2011; Suhail et al., 2019). Yet, the majority of currently available therapeutics do not inhibit metastasis, and only target the primary tumor where the cancer initially arises from.

To screen anti-cancer drugs, researchers often carry out experiments on mice or cells grown in the laboratory. While these model systems have led to effective treatments, they have limitations when it comes to testing drugs that block metastasis. For instance, cells cultured in the laboratory cannot accurately replicate tumor progression in humans (Katt et al., 2016), and metastasis can take at least several weeks to appear in mouse models, which are expensive to create and maintain (Simons and Brayton, 2017). Now, in eLife, Joji Nakayama, Zhiyuan Gong and co-workers report an innovative zebrafish model for screening anti-metastasis drugs (Nakayama et al., 2021).

The zebrafish was introduced to the research field in 1972, and has become a powerful model system for cancer research, due to its relative transparency, high reproduction rates, and genetic similarity to humans (Brown et al., 2017; Chen et al., 2021; Fazio et al., 2020; Gamble et al., 2021; Hason and Bartůněk, 2019). Early in development, cells in the zebrafish embryo undergo a morphological change and migrate inwards via a process called epiboly (Bruce and Heisenberg, 2020). The way these healthy cells move is similar to how cancer cells travel across tissues during metastasis. Hence, Nakayama et al. proposed that small-molecule inhibitors that interrupt epiboly may also suppress metastasis.

The team (who are based at the National University of Singapore, the National Cancer Center in Japan and other institutes in Singapore and Japan) found that some of the genes expressed during zebrafish epiboly are also activated during tumor metastasis. This finding provides the experimental support that zebrafish epiboly can serve as a model for tumor cell movement. So, Nakayama et al. developed a zebrafish screening platform, which they used to test 1,280 drugs that had already been approved by a government agency, such as the US Food and Drug Administration (FDA) or the European Medicines Agency (EMA).

The screen was carried out on zebrafish embryos exposed to a specific drug at four hours post-fertilization (Figure 1A). Nakayama et al. found that 132 of the drugs tested induced a delay in epiboly after five hours of treatment. Several of these drugs had previously been reported to inhibit molecular mechanisms associated with metastasis (Liu et al., 2013; Nakayama et al., 2020).

**Figure 1.**
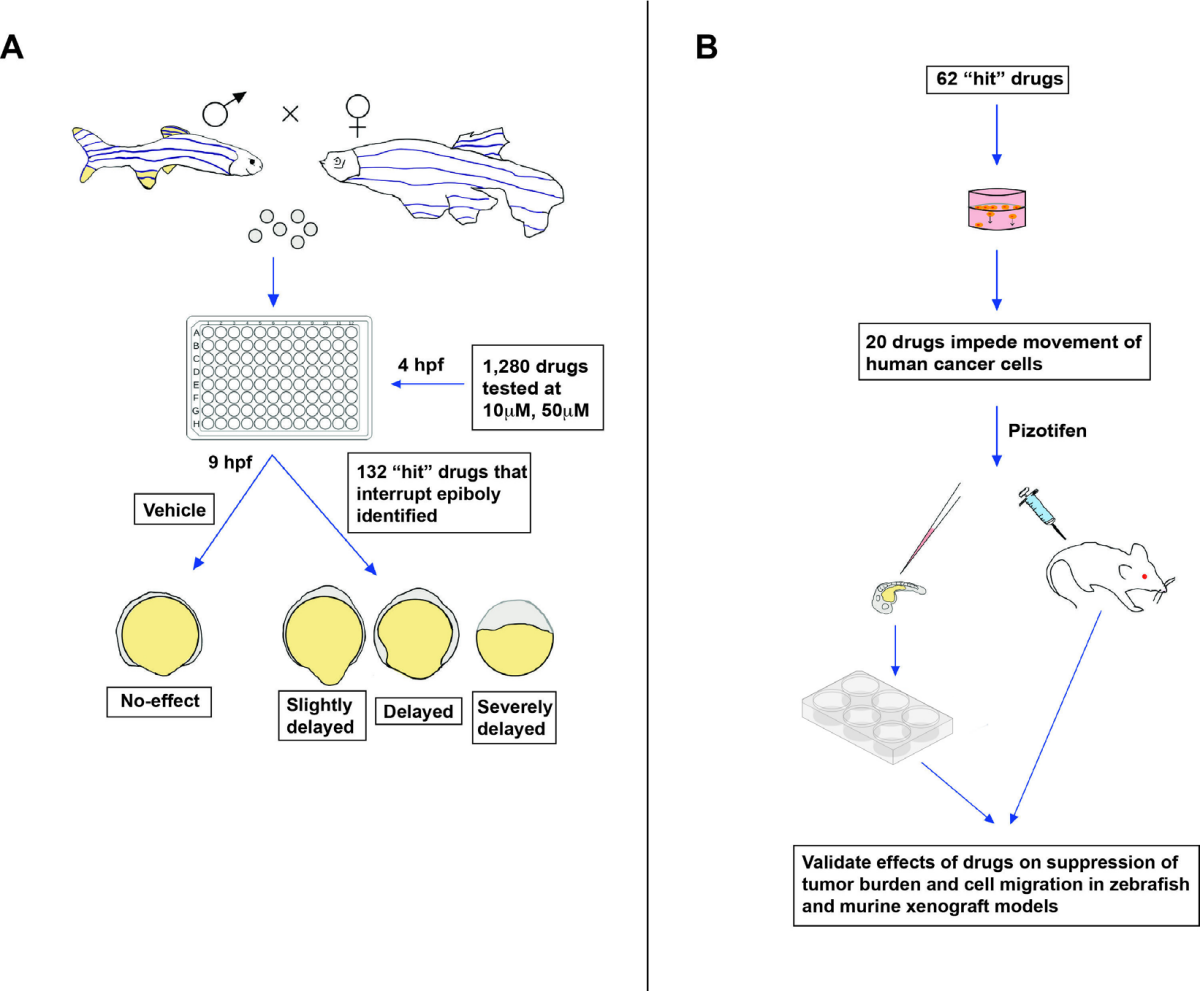
New approach for screening anti-metastasis drugs. (**A**) Zebrafish were bred and their embryos were collected and plated into individual wells. Each embryo was treated four hours post-fertilization (hpf) with either the vehicle (an inactive substance that the drug is mixed with to facilitate administration) or a drug that had been approved by the FDA, EMA, or another government agency for cancer treatment: two concentrations were administered (10 µm and 50 µm). Out of the 1,280 drugs tested, 132 interrupted or delayed epiboly five hours after the drug was administered. (**B**) 62 of these positive hits were then tested on tumor cells cultured in the laboratory. This revealed that 20 of these drugs also impeded the migration of cancer cells, in addition to disrupting epiboly in zebrafish. One of the identified drugs, called Pizotifen, was then administered to zebrafish and mice models that had been injected with fluorescently labelled human cancer cells (commonly referred to as xenografts). This showed that the drug can also suppress metastasis in vivo.

Image credit: Emily Hill; the 6-well plate is adapted from an image by DataBase Center for Life Science (DBCLS; CC BY 4.0).

Nakayama et al. then used cell-based assays to test whether 62 of these 132 ‘positive hits’ (which also delay epiboly in vitro) can suppress the migration of cancer cells (Figure 1B). The tumor cells were placed in a chamber with or without the drug, and the team recorded how many could crawl into the neighboring compartment after a few hours of treatment. This revealed that 20 of the drugs that disrupted epiboly also impeded the movement of human cancer cells.

Finally, Nakayama et al. tested if one of the epiboly-interrupting drugs called Pizotifen could also impair tumor cell movement in living animals: this drug was selected because its primary target (serotonin receptor 2C) is highly expressed in human cancer cells during metastasis. To do this, they injected fluorescently labelled cancer cells into zebrafish embryos, and found that fish exposed to Pizotifen experienced significantly less metastasis than fish treated with a placebo. Similar observations were made in mice that had cancer cells injected into their breast-like tissue, half of which were treated with a daily dose of Pizotifen, and half of which received a placebo (Figure 1B).

The screening platform created by Nakayama et al. makes it easy to rapidly find new drugs that suppress metastasis, while circumventing the limitations of cell culture and mouse model systems. In addition, zebrafish injected with human cancer cells can serve as an additional means for narrowing down which drugs to test in mouse models. Having zebrafish join the drug discovery platform will hopefully result in more and better treatments for patients with metastatic cancers.

## References

[bib1] Brown HK, Schiavone K, Tazzyman S, Heymann D, Chico TJ (2017). Zebrafish xenograft models of cancer and metastasis for drug discovery. Expert Opinion on Drug Discovery.

[bib2] Bruce AEE, Heisenberg CP (2020). Mechanisms of zebrafish epiboly: a current view. Current Topics in Developmental Biology.

[bib3] Chaffer CL, Weinberg RA (2011). A perspective on cancer cell metastasis. Science.

[bib4] Chen X, Li Y, Yao T, Jia R (2021). Benefits of zebrafish xenograft models in cancer research. Frontiers in Cell and Developmental Biology.

[bib5] Fazio M, Ablain J, Chuan Y, Langenau DM, Zon LI (2020). Zebrafish patient avatars in cancer biology and precision cancer therapy. Nature Reviews Cancer.

[bib6] Gamble JT, Elson DJ, Greenwood JA, Tanguay RL, Kolluri SK (2021). The zebrafish xenograft models for investigating cancer and cancer therapeutics. Biology.

[bib7] Hason M, Bartůněk P (2019). Zebrafish models of cancer-new insights on modeling human cancer in a non-mammalian vertebrate. Genes.

[bib8] Katt ME, Placone AL, Wong AD, Xu ZS, Searson PC (2016). In vitro tumor models: advantages, disadvantages, variables, and selecting the right platform. Frontiers in Bioengineering and Biotechnology.

[bib9] Liu P, Kumar IS, Brown S, Kannappan V, Tawari PE, Tang JZ, Jiang W, Armesilla AL, Darling JL, Wang W (2013). Disulfiram targets cancer stem-like cells and reverses resistance and cross-resistance in acquired paclitaxel-resistant triple-negative breast cancer cells. British Journal of Cancer.

[bib10] Nakayama J, Lu JW, Makinoshima H, Gong Z (2020). A novel zebrafish model of metastasis identifies the HSD11β1 inhibitor adrenosterone as a suppressor of epithelial-mesenchymal transition and metastatic dissemination. Molecular Cancer Research.

[bib11] Nakayama J, Tan L, Li Y, Goh BC, Wang S, Makinoshima H, Gong Z (2021). A zebrafish embryo screen utilizing gastrulation identifies the HTR2C inhibitor Pizotifen as a suppressor of EMT-mediated metastasis. eLife.

[bib12] Simons BW, Brayton C, Simons BW (2017). Patient Derived Tumor Xenograft Models: Promise, Potential and Practice.

[bib13] Suhail Y, Cain MP, Vanaja K, Kurywchak PA, Levchenko A, Kalluri R, Kshitiz ­ (2019). Systems biology of cancer metastasis. Cell Systems.

